# Game Utilization and Performance Following RTP From ACL Reconstruction Does not Influence a Subsequent Second ACL Injury in National Football League Players

**DOI:** 10.1016/j.asmr.2021.07.002

**Published:** 2021-07-29

**Authors:** Lafi S. Khalil, Kevin G. Lindsay-Rivera, Muhammad J. Abbas, Sabin Shah, Marissa Tandron, Albert Ferris, Kelechi R. Okoroha

**Affiliations:** a Henry Ford Hospital, Department of Orthopaedic Surgery, Detroit, Michigan, U.S.A.; b Wayne State University, School of Medicine, Detroit, Michigan, U.S.A.; c Mayo Clinic, Department of Orthopedic Surgery, Minneapolis, Minnesota, U.S.A.

**Keywords:** ACL rerupture, anterior cruciate ligament, National Football League, reinjury, retear

## Abstract

**Purpose:**

The purpose of this study is to evaluate differences in game utilization or performance following primary anterior cruciate ligament (ACL) reconstruction between National Football League (NFL) players with and without a second ACL injury.

**Methods:**

NFL players who underwent ACL reconstruction between 2013 and 2017 were identified. Players were classified as having one injury (“tear”) or having later sustained a subsequent second (reinjury or contralateral) ACL injury (“retear”). Players were excluded if they tore their ACL prior to the NFL, did not return to play (RTP), did not play the season before injury, or had concomitant injuries. Demographic characteristics, game utilization statistics, and season approximate value (SAV) performance metrics were recorded. Statistical analysis compared data after RTP from primary ACL reconstruction (seasons +1, +2, and +3) relative to the season before injury (season −1) between cohorts using mean differences and relative percentages.

**Results:**

Analysis included a total of 45 players, 32 in the “tear” group and 13 in the “retear” group. Demographics, level of play, and time to RTP after primary ACL reconstruction did not differ between the groups (*P* > .05). Tear and retear groups demonstrated similar utilization and performance metrics the season prior to injury (−1) and the 3 seasons following RTP (season of injury is “0”). Both groups had a similar decrease (relative percentage) in games played and started, snap counts, and SAV during the 3 seasons following RTP compared to baseline (*P* > .05). The draft pick position was correlated with the relative percentage of games started the first season after RTP (*r* = .6, *P* = .02).

**Conclusions:**

Game utilization and performance metrics following ACL reconstruction were not associated with a subsequent second ACL injury. Players with a higher draft pick position were more likely to return to the starting lineup following primary ACL reconstruction. Ultimately, player game utilization and performance following primary ACL reconstruction is not predictive of a subsequent second ACL injury.

**Level of Evidence:**

Level III, retrospective case-control study

## Introduction

Anterior cruciate ligament (ACL) ruptures are one of the most prevalent knee injuries in sports with an estimated annual incidence of 200,000 nationally.^1^^,^^2^ National Football League (NFL) players are at an increased risk of ACL injuries compared to the general population due to the strenuous demands placed on the knee with frequent jumping, collisions, pivoting, and cutting maneuvers.^3^ Following ACL reconstruction, NFL players have demonstrated a high rate of return to play (RTP): 63% among linemen,^4^ 79% among wide receivers and running backs,^5^ 92% of quarterbacks,^1^ and 63% overall.^6^ Given the prevalence of NFL athletes with a history of ACL injury, it is paramount to identify factors potentially contributing to a second ACL injury in this population.

Among NFL athletes entering the NFL with a history of ACL reconstruction, there is an approximately 25% incidence of players who sustain a second ACL injury during their NFL career.^3^^,^^7^ When considering athletes overall following an ACL injury, prior literature suggests that the rate of retear or reinjury requiring a revision reconstruction can be up to 6 times higher in a group of athletes that had already underwent an ACL reconstruction compared with healthy controls.^8^ Additionally, athletes with a history of ACL reconstruction have a higher likelihood of suffering a contralateral ACL injury.^7^, ^8^, ^9^ The work of Paterno et al. identified the psychological toll of a primary ACL injury and subsequent reconstruction as a significant factor in increasing the risk of a second ACL tear,^10^ while the fear of suffering a reinjury, in turn, places a great psychological toll on athletes as well.^11^^,^^12^ Given the decrease in average career lifespan of NFL players to approximately 3 seasons,^13^ from 4.6 seasons a decade ago,^14^ the potential of an ACL injury to negatively affect the longevity and performance of an NFL career can be a concern for athletes and organizations.^6^^,^^15^, ^16^, ^17^ While the risk of a subsequent second ACL injury is certainly multifactorial, there has been limited evaluation on how the in-game utilization of players returning from primary ACL reconstruction can influence the incidence of a subsequent second ACL tear in elite NFL athletes.

There is currently a paucity of studies examining the effects of game utilization and participation following RTP from ACL reconstruction in NFL athletes as a modifiable risk factor for a subsequent second ACL injury. The purpose of this study is to evaluate differences of in game utilization or performance following primary ACL reconstruction between NFL players with and without a second ACL injury. The authors hypothesize that no differences in game utilization or performance following primary ACL reconstruction exist between NFL players with and without a subsequent ACL injury to either their ipsilateral or contralateral knee.

## Methods

The authors conducted a retrospective study of ACL ruptures sustained by all NFL players from 2012/2013 season to the 2017/2018 season. Players who underwent ACL reconstruction were identified using publicly available data, such as website searches, injury reports, and team websites, using methods similar to previous studies.^1^^,^^4^^,^^18^, ^19^, ^20^, ^21^, ^22^ Additionally, players who sustained a second ACL rupture and required either a revision ACL reconstruction or a primary ACL reconstruction on the contralateral knee were identified. Injuries were confirmed using a minimum of two independent publicly available sources, which corroborate the injury. Dates of surgery were verified and cross referenced with gaps in statistical input and with team roster moves.

**Fig 1:**
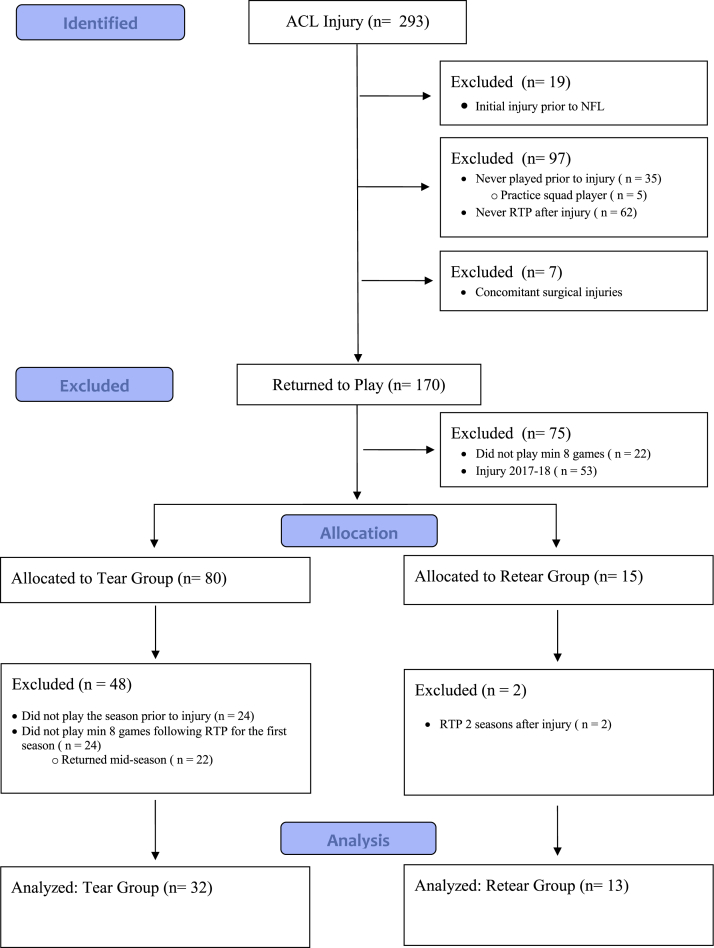
Flow diagram of player inclusion for tear and retear groups. ACL, anterior cruciate ligament; min, minimum; NFL, National Football League; RTP, return to play.

The control (“tear”) group comprised NFL athletes who underwent a primary ACL reconstruction during the study period. The study (“retear”) group comprised NFL players who underwent an initial ACL reconstruction and successful RTP, but subsequently sustained a second ACL injury to either the contralateral or ipsilateral knee. This study constitutes a case-control study of players with a primary ACL reconstruction who did and did not sustain a second ACL injury. Players were excluded from the study if any of the following conditions were met; because of missing data points, players were excluded if initial ACL injury occurred prior to entering the NFL or if they did not play the season prior to ACL injury. Additionally, a history of ACL tear prior to the NFL has been shown to negatively influence early career utilization and performance compared to healthy controls, and therefore, these players were excluded to reduce bias.^23^ Additionally, players were excluded if they retired or never returned to play after ACL reconstruction, or if they did not play a minimum of 8 games (half a season) during the first full season after RTP and did not play any further seasons thereafter. Other exclusion criteria included if their injury occurred as recent as the 2017/2018 season but their RTP game data were not yet complete enough for inclusion (minimum of 8 games/half a season) or if they had concurrent or alternate injuries such as patellar tendon ruptures, Achilles tendon ruptures, multiligamentous knee injuries, or contralateral injuries requiring surgery (Fig 1).

Data collection methods for all players included height, weight, body mass index, age at time of injury, position, season of injury, week of injury within the season, quarter of injury within the game, starter versus bench player role (according to depth chart position) at the time of injury, time to RTP, and all reported official NFL combine performance measures and draft statistics.^24^ Additionally, game utilization and performance metrics were documented from football-specific statistical websites, such as pro-football-reference.com, ESPN.com, and NFL.com, and cross-referenced for validation. Game data were collected for the season prior to injury (season −1), the season of injury (index season), and the first 3 seasons following RTP (seasons 1, 2, and 3). The main performance metric used is the season approximate value (SAV) calculation, which is determined by pro-football-reference.com and is a numerical calculation of the relative contribution each athlete makes toward their teams’ success. This calculation is based on a different formula for each position; therefore, no single formula exists, but the reported SAV is instead normalized across positions in order to provide a standardized metric to compare athletes across positions and has been used in prior literature. The SAV is given to players at any position—weighing the relative contribution of different more commonly recorded statistics for a particular position—and generates a value that correlates to their overall helpfulness to a team in that year and has been analyzed in previous literature.^19^^,^^25^

Game utilization and performance variables were analyzed to evaluate changes from the baseline season before injury (season −1) relative to the 3 seasons following RTP. For a complete analysis, the control and study groups were compared for game utilization and performance in several manners. First, each season was compared between groups. Additionally, changes from baseline (relative to season −1) in seasons 1 through 3 following RTP were compared between the two groups. Lastly, relative percentages were calculated using the season prior to injury (season −1) as the benchmark for each individual season following RTP, similar to a prior study.^19^ Relative percentages for seasons 1, 2 and 3 following RTP are either greater than 100% if the game utilization or performance variable exceeds that of the season prior to injury, or less than 100% if it fails to meet baseline. This allows an individualized and normalized comparison between groups irrespective of differences in baseline game utilization and performance. Given the short career duration of NFL athletes on average,^13^^,^^14^ a lengthier career assessment was not undertaken as the sample size was insufficient.

### Statistical Analysis

Continuous data were reported as means ± SD, while categorical data were reported as counts and percentages (N [%]). For continuous variables, univariate two-group comparisons were performed using independent 2-sample *t*-tests if the variable was normally distributed, and Wilcoxon rank sum tests were used if the variable were non-normally distributed. For categorical variables, univariate two-group comparisons were performed using χ^2^ tests when expected cell counts were >5, and using Fisher’s exact tests when expected cell counts were <5. Repeated-measures analyses were performed to see whether the performance variables changed differently over time within groups. For repeated-measures analyses, data were reported as adjusted means (standard errors) and compared between groups as well. Relative percentages were calculated for each of the 3 seasons after injury, using preinjury season 1 as the baseline. Correlation coefficients were performed between relative percentages and combine performance variables. Statistical significance was set at *P* < .05. All analyses were performed using SAS 9.4 (SAS Institute Inc., Cary, NC).

## Results

### Demographics

During the study period, 293 ACL injuries occurred in the NFL, of which 45 NFL players, with available game data prior to and following return from primary ACL reconstruction, met inclusion criteria (Fig 1). Thirty-two athletes sustained only primary injury and were in the “tear” group, while 13 players suffered a reinjury (ipsilateral or contralateral) and were in the “retear” group. Table 1 illustrates the demographic characteristics of both cohorts. The tear and retear groups had a mean age (± SD) of 25.9 ± 2.9 and 26.6 ± 3.3 years, and body mass index of 32.0 ± 4.7 and 32.7 ± 5.3 kg/m^2^, respectively (*P* > .05). Numbers of seasons before injury were 3.19 ± 2.8 and 3.15 ± 2.9 for the tear and retear groups (*P* > .05). There were no significant differences in positions played or starter versus bench player roles between groups. However, 122 of the 293 (41.6%) ACL injuries initially identified, and 15 of the 45 (33.3%) included in the final analysis, occurred in “speed” position players (wide receivers, linebackers, running backs, and tight ends). The timing of the first ACL injury relative to the season was evenly distributed. Of the 13 players in the retear group, 7 (54%) sustained ACL reruptures of the ipsilateral knee, and 6 (46%) sustained an ACL injury to the contralateral knee. There was no significant difference between the tear and retear group in the average number of months to RTP after primary ACL reconstruction. The NFL combine performance metrics for players from both groups were not statistically different. Of the 293 ACL injuries discovered during the study period, it was noted that 19 players had a history of an ACL injury prior to the NFL, 35 players were rostered on a team but never played in an NFL game prior to injury, and 7 players had concomitant injuries. Therefore, the return to play rate among the remaining 232 players was 73.3% (170/232) following primary ACL reconstruction in active NFL players in the present cohort.

**Table 1:** Demographic and Utilization Characteristics at Time of Primary Anterior Cruciate Ligament Injury

	Tear	Retear	*P* Value
Number of players	32 (71%)	13 (29%)	
Age (years)	25.9 (2.9)	26.6 (3.3)	.457
Height (inches)	73.9 (2.6)	74.5 (2.9)	.448
Body mass index (kg/m^2^)	32.0 (4.7)	32.7 (5.3)	.673
Position			.676
Quarterback	1 (3%)	2 (15%)	
Running back/Fullback	2 (6%)	0 (0%)	
Wide receiver	7 (22%)	1 (8%)	
Tight end	1 (3%)	0 (0%)	
Offensive lineman	7 (22%)	4 (31%)	
NT/DL	5 (16%)	1 (8%)	
Defensive end	4 (13%)	1 (8%)	
Linebacker	2 (6%)	2 (15%)	
Cornerback/Safety	3 (9%)	2 (15%)	
Start/Bench			.173
Starter	15 (47%)	9 (69%)	
Bench	17 (53%)	4 (31%)	
Time of injury			.969
Preseason	8 (25%)	4 (31%)	
Weeks 1-4	4 (13%)	1 (8%)	
Weeks 5-8	9 (28%)	2 (15%)	
Weeks 9-12	3 (9%)	2 (15%)	
Weeks 13-16	4 (1%)	2 (15%)	
Playoffs	2 (6%)	1 (8%)	
Offseason	2 (6%)	1 (8%)	
SBI (seasons)	3.19 (2.82)	3.15 (2.91)	.971
Injured leg (first tear)			.141
Right	20 (63%)	5 (38%)	
Left	12 (38%)	8 (62%)	
Injured leg (second tear)			
Ipsilateral		7 (53.9%)	
Contralateral		6 (46.1%)	
RTP primary (months)	11.61 (2.70)	10.36 (1.75)	.162
RTP revision (months)		19.10 (25.92)	
Combine performance			
Draft pick	102.2 (65.1)	83.3 (86.7)	.211
40 yard dash (s)	4.76 (.31)	4.84 (.33)	.48
Bench press (reps)	22.2 (8.5)	24.2 (10.2)	.592
Broad jump (in)	113.3 (7.6)	110.9 (10.0)	.472
Shuttle drill (s)	4.43 (.29)	4.47 (.30)	.738
3 cone drill (s)	7.20 (.40)	7.34 (.40)	.404
Vertical (in)	32.6 (3.9)	31.9 (3.4)	.635

Continuous variables are presented using mean (SD). Categorical variables are presented using frequency (percentage). NT/DL, nose tackle/defensive lineman; RTP, return to play; SBI, number of seasons before injury.

### Utilization and Performance by Season

Table 2 illustrates utilization variables of games played (GP), games started (GS), and average snaps played per game (Snaps), as well as the standardized performance metric SAV. There were no significant differences (*P* > .05) in utilization and performance metrics between tear and retear groups the season prior to injury (season −1) or the 3 seasons following RTP from primary ACL reconstruction (seasons +1, +2, and +3). When cohorts were stratified by positions, no significant differences in utilization or performance metrics existed between cohorts for offensive (*P* > .05) or defensive (*P* > .05) players.

**Table 2:** Game Utilization Trends by Season: Before and After Primary Anterior Cruciate Ligament Reconstruction

Variable	Seasons before and after Injury	Tear (*n* = 32)	Retear (*n* = 13)	*P* Value: All Players	*P* Value: Offensive Players	*P* Value: Defensive Players
GP	−1	13.96 (3.09)	13.91 (2.59)	.798	.778	.953
	Index	7.45 (4.37)	10.13 (4.42)	.165	.204	.549
	+1-3 Avg	11.48 (4.39)	10.69 (3.67)	.415	.318	.770
GS	−1	11.17 (5.16)	11.00 (5.44)	.984	.454	.371
	Index	6.76 (4.70)	8.88 (4.88)	.28	.558	.71
	+1-3 Avg	9.37 (5.43)	7.44 (4.59)	.303	.483	.394
Snaps	−1	704.1 (330.5)	865.2 (189.3)	.414	.516	.523
	Index	393.9 (303.9)	499.1 (287.2)	.296	.671	.447
	+1-3 Avg	539.59 (337.54)	519.06 (291.12)	.749	.764	.963
SAV	−1	6.78 (5.05)	6.09 (4.39)	.746	.584	.427
	Index	2.91 (2.20)	5.13 (4.45)	.162	.259	.441
	+1-3 Avg	4.69 (4.16)	4.03 (3.22)	.861	1.000	.1000

GP, games played; GS, games started; Snaps, number of snaps played; SAV, season approximate value. Seasons +1-3 Avg, the weighted average of each variable over the first 3 seasons following RTP. Continuous variables are presented using mean (SD).

### Utilization and Performance Relative to Baseline

Following RTP, changes in each utilization and performance variable relative to the season prior to injury (season −1) were calculated as adjusted mean differences for seasons +1, +2, and +3 (Table 3). Athletes in both the tear and retear group demonstrated decreased GP, GS, Snaps, and SAV during seasons +1, +2, and +3, as compared to baseline (season −1), albeit not statistically significant (*P* > .05 for all seasons). Decreases in utilization and performance variables during each of the 3 seasons following RTP from the first ACL injury were statistically similar between the tear and retear groups (*P* > .05). Simple logistic regression analysis demonstrated that no utilization or performance variables following primary ACL reconstruction were significantly predictive of whether the second injury occurred on the ipsilateral or contralateral knee in the retear group (*P* > .05).

**Table 3:** Adjusted Mean Differences Following RTP Relative to the Season Before Injury

Variable	Season after RTP	Tear	Retear	*P* Value‡
GP	+1	−1.93 (1.17)	−2.45 (1.92)	.653
	+2	−2.5 (1.24)	−4.21 (1.96)	.461
	+3	−3.3 (1.37)	−3.74 (2.28)	.872
	*P* value∗	>.05	>.05	
GS	+1	−1.01 (1.58)	−4.5 (2.42)	.265
	+2	−1.34 (1.7)	−1.14 (2.67)	.502
	+3	−1.11 (1.8)	−2 (2.96)	.363
	*P* value∗	>.05	>.05	
Snaps	+1	−133.79 (91.08)	−306.28 (183.7)	.467
	+2	−183.39 (96.74)	−392.5 (183.7)	.153
	+3	−182.26 (109.96)	−315 (201.23)	.585
	*P* value∗	>.05	>.05	
SAV	+1	−1.68 (1.19)	−1.84 (1.89)	.929
	+2	−1.81 (1.23)	−2.59 (1.97)	.268
	+3	−1.33 (1.37)	−.23 (2.18)	.591
	*P* value∗	>.05	>.05	

GP, games played; GS, games started; RTP, return to play; SAV, season approximate value; Snaps, number of snaps played. Adjusted mean difference (standard error) of each variable during seasons 1-3 relative to the season prior to injury (season −1). Adjusted mean differences for seasons +1, +2, and +3 are compared relative to the season prior to injury (−1)

∗ Within each group, with all 3 seasons having nonsignificant *P* values.

‡ Adjusted mean differences compared between groups.

### Utilization and Performance Relative Percentages

**Fig 2:**
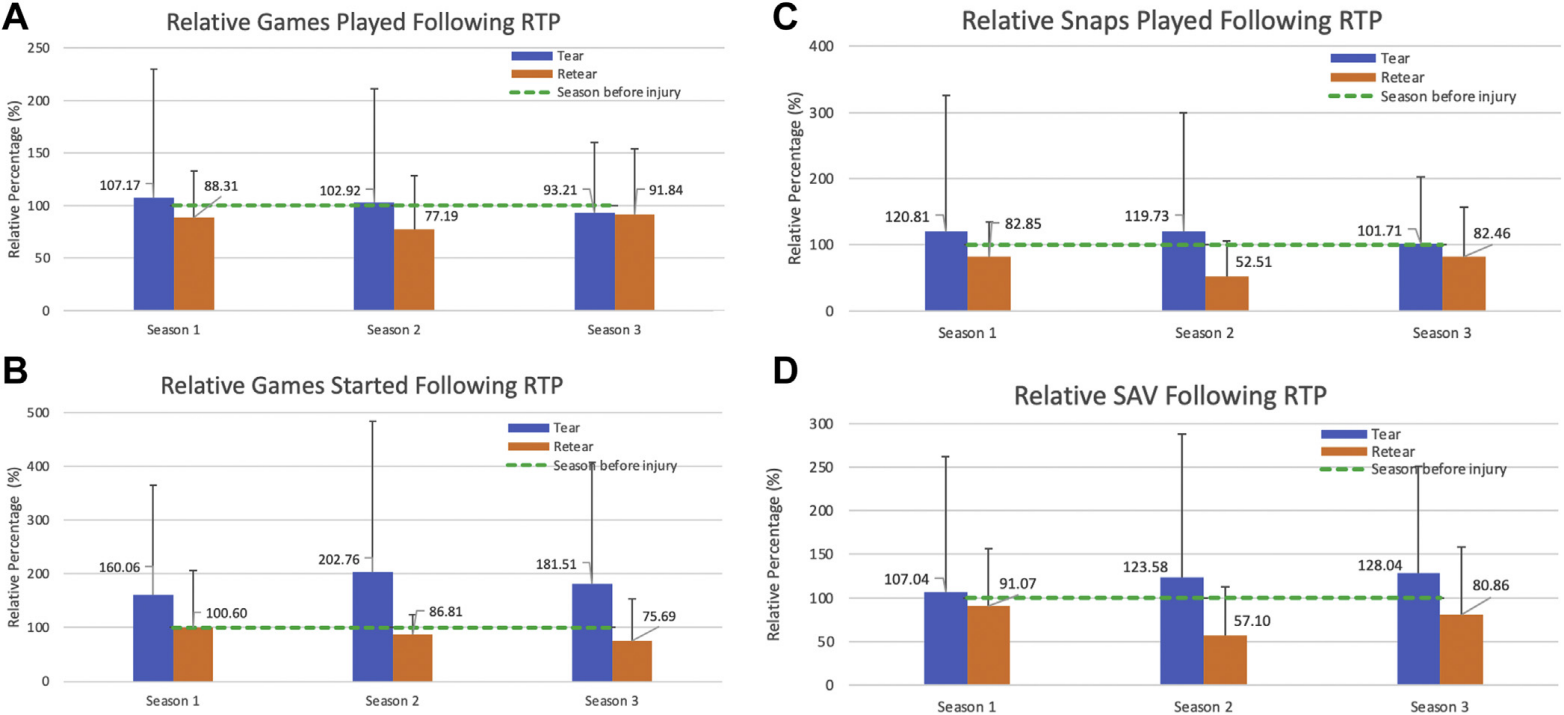
Relative percentage of games played (A), games started (B), snaps played (C), and season approximate value (SAV) (D) during the first three seasons following return to play (RTP) from anterior cruciate ligament reconstruction among tear and retear players. Preinjury baseline is represented by the dotted horizontal line (100%) across all variables, which corresponds to the season prior to injury. Relative percentages below this line indicate that the post-index variable was less than baseline, while values above this line indicate an increase in post-index variable relative to baseline. ∗No significant differences (*P* < .05) exist between tear and retear groups.

Using the season prior to injury as baseline, relative percentages were calculated for seasons +1, +2, and +3 following RTP from the index ACL injury for each utilization and performance variable (Fig 2). Players in the tear cohort exceeded 100% of their baseline metrics for GP, GS, Snaps, and SAV in all 3 seasons following ACL reconstruction, except for GP during season +3 (93.2%). Players in the retear cohort failed to achieve 100% of their baseline metrics for GP, GS, Snaps, and SAV during any of the first 3 seasons following RTP from their first ACL reconstruction, except for GS during season +1 (100.6%). There were no statistically significant differences in relative percentages for any utilization or performance metric compared between tear and retear groups over the first 3 seasons following RTP (*P* > .05).

### Utilization and Performance Association with Draft Position

Table 4 displays the Pearson correlation coefficients between NFL draft combine metrics and the relative percentages for each utilization and performance metric during the first 3 seasons following RTP for all players in the tear and retear groups. A positive, medium-strength correlation was found between the draft pick number and the relative percentage of GS 1 season after RTP (GS +1: *r* = .554; *P* = .017). Otherwise, there were no significant correlations found between NFL combine performance and utilization and performance metrics following RTP from primary ACL reconstruction.

**Table 4:** Pearson Correlations Between Relative Percentages Per Season Following Return to Play with National Football League Combine and Draft Performance

Relative %	Season	Draft Pick	40-Yard Dash	Bench Press	Broad Jump	Shuttle Drill	3-Cone Drill	Vertical Jump
GP	+1	−.05905	.18312	−.05817	−.13112	.05257	−.02138	−.08522
	+2	−.2902	.16458	−.09883	−.20308	−.0521	−.00786	−.07397
	+3	−.24005	.16444	−.04761	−.18193	.00902	.05111	−.16958
GS	+1	**.55365**	−.01877	−.06586	.03925	−.20062	−.26434	.0818
	+2	.08278	−.14673	−.08272	−.08949	−.22439	−.3256	.19003
	+3	.4081	−.13769	.00555	−.0515	−.39057	−.3389	.1771
Snaps	+1	.23361	.1568	−.11643	−.11833	.03247	−.07479	−.07056
	+2	−.0865	.05237	−.31587	−.13145	−.07008	−.07561	.02423
	+3	−.32457	.18144	−.08385	−.16872	−.09243	−.09267	−.15369
SAV	+1	−.19553	.1847	−.00445	−.18584	−.02145	−.08167	−.06645
	+2	−.08737	.04801	−.1055	−.10953	−.12522	−.14765	.04431
	+3	.1457	−.09438	−.22935	−.07532	−.34645	−.27418	.18729

National Football League combine and draft performance correlations with the relative percentages of games played (GP), games started (GS), snaps played, and season approximate value (SAV) during seasons 1 through 3 following return to play from primary anterior cruciate ligament reconstruction relative to the season before injury (season −1). Pearson correlation coefficients expressed as Prob > |*r*| under H0: Rho = 0. Significant values are noted in bold; *P* < .05.

## Discussion

Among NFL players who played at least 1 season in the NFL prior to their primary ACL reconstruction, there were no significant differences in game utilization and performance upon RTP between players who never sustained a subsequent second ACL tear (tear players) and those who did (retear players). Although tear players returned to baseline game utilization and performance following ACL reconstruction, averaging greater than 100% relative percentages during the first 3 seasons, this was not statistically different than retear players who predominantly failed to achieve baseline markers. This finding highlights that game utilization and performance, as surrogate measures of overall athletic demand following primary ACL reconstruction, are not predictive of another ACL injury. Interestingly, players with a higher NFL draft pick position demonstrated a significant correlation with the relative percentage of games started following RTP from primary ACL reconstruction. Overall, 73.3% of NFL players were able to RTP following primary ACL reconstruction.

The present study sought to determine whether game utilization and performance following RTP from primary ACL reconstruction was a significant predictive factor among NFL players who subsequently sustained a second ACL injury compared to those who did not. The presented results demonstrated no significant differences in snaps, GS, or GP, and the amalgamation statistic of value (SAV) to team. When analyzing the data based on subgroups, such as position of the player, a similar lack of significance was seen. These results echo the findings of Okoroha et al., who evaluated time to RTP following primary ACL reconstruction and found it not to be a risk factor of sustaining a reinjury.^26^ Likewise, Cinque et al. compared NFL linemen who RTP following primary ACL reconstruction to a healthy, matched control group, demonstrating no significant differences in game performance or career longevity between the two cohorts.^4^ Despite the encouraging findings following ACL reconstruction in the previously mentioned studies, neither evaluated game utilization and performance as a risk factor for ACL reinjury.

The higher risk of reinjury in NFL athletes who have had a prior ACL injury is well established in the literature.^3^^,^^8^ The work of Dodson et al. demonstrates that nearly one-fifth (18.3%) of ACL injuries sustained were in fact the players’ second ACL injury, with 12.3% retears in players that had previously undergone an ipsilateral ACL reconstruction and 7.3% tears to the contralateral knee.^3^ In a review of NFL combine participants with a prior ACL injury, Connor et al. found a 25% incidence of a subsequent second ACL injury (12 ipsilateral and 14 contralateral) during their NFL career, which was significantly greater than the incidence of primary ACL injury among a healthy, matched control cohort (18/200 = 9%; *P* < .001).^7^ The results from the present study demonstrated that from a cohort of NFL athletes who played in NFL games prior to their first ACL reconstruction and successfully returned to play for at least half of an NFL season, 28.9% (13/45) sustained a second ACL injury, with approximately equal distribution of reinjury and contralateral injury (54% vs 46%). Although these results show a greater incidence of subsequent injury than prior literature, it is likely secondary to the stringent inclusion criteria applied. This was done intentionally to limit potentially confounding factors introduced by heterogenous data of players with no prior NFL experience or significant RTP time after injury.

In evaluating player factors contributing to injury, Dodson et al. determined that most players who sustained a second ACL injury were in so-called “speed” positions, including wide receivers, linebackers, running backs, and tight ends.^3^ In fact, most NFL-caliber athletes who sustain an ACL tear do so through a noncontact mechanism. Retrospective video analyses revealed that 72% of ACL injuries in the NFL occur with a pivoting motion, as is often exhibited by a “speed” player who places a large valgus moment on the affected extremity at the knee.^27^ In the present study, 122 of the 293 (41.6%) ACL injuries initially identified, and 15 of the 45 (33.3%) included in the final analysis, occurred in speed position players. As this biomechanical, noncontact pivoting mechanism may be related to fatigue from overuse and neuromuscular deconditioning,^28^^,^^29^ it is prudent to continue evaluating whether “load management” strategies are effective in reducing the risk of reinjury.^30^^,^^31^ Given the prevalence of ACL injury in NFL players and the high degree of collisions that place relatively large stresses on the reconstructed knee,^11^^,^^32^, ^33^, ^34^ there has been a concerted effort to address a player’s return to full-time duty and determine appropriate game utilization while minimizing reinjury.^1^^,^^4^^,^^26^^,^^35^^,^^36^

The present results did indicate that players picked with higher draft positions were significantly more likely to start in games the season after RTP from ACL reconstruction. This phenomenon parallels findings from a prior study by Okoroha et al., which investigated RTP following revision ACL reconstruction and determined that NFL athletes with higher draft positioning or greater NFL experience prior to revision surgery were more likely to RTP.^35^ Daruwalla et al. similarly demonstrated that college players higher in the depth chart were more likely to RTP following ACL reconstruction.^37^ It is worth noting that the current study excluded players without prior NFL experience, and both tear and retear groups had similar NFL experience prior to their first ACL injury. One strength of this study is the novelty in comparing a cohort of athletes who sustained their index ACL injury while in the NFL, thereby enabling objective evaluation of their game utilization and performance upon RTP relative to their NFL preinjury baseline utilization and performance, compared with those later sustaining a second ACL injury. The finding of draft pick position influencing rates of RTP and GS highlights the competitive nature of an NFL career and the potential for future studies to evaluate whether draft pick position and game performance may, in fact, be greater predictors of career longevity than a history of ACL reconstruction or subsequent reinjury.

### Limitations

This study, as with other retrospective studies, has limitations in the form of potential bias and confounding variables impacting the results of the study. Largely, these biases were introduced in the data collection as an Internet-based review of statistics websites. Nevertheless, the methodology closely resembled that of prior studies.^1^^,^^4^^,^^18^, ^19^, ^20^, ^21^, ^22^ Similarly, these data and conclusions cannot be applied to a more general population of athletes or for other levels of play in American football such as college, high school, or youth football, as the present cohorts specifically sustained their primary injury while in the NFL. Furthermore, the classification and grading of injuries, and other concomitant injuries, such as meniscal and cartilage damage, was not possible due to the lack of availability of official medical records and imaging for review. Likewise, surgical technique, graft selection, fixation methods, and postoperative rehabilitation protocols were not standardized. As the NFL is a competitive league and game decisions are made by coaching staff and roster composition, some factors may confound game utilization and performance for which we cannot account. Because of the lack of publicly reported statistical data prior to the study period (2012), the sample size is too small to independently analyze players with ipsilateral and contralateral reinjuries; therefore, the current study is unable to predict the risk of ipsilateral or contralateral reinjury independently. A power analysis was not conducted, as all players with available statistics since 2012 were eligible for inclusion. Future studies with larger sample sizes are required to determine these differences. Exclusion of NFL athletes with a history of ACL injury prior to entering the NFL greatly limited the sample size of the present study, but it prevented the analysis of heterogenous data as all included players’ game data were compared chronologically relative to the index season.

### Conclusion

Game utilization and performance metrics following ACL reconstruction were not associated with a subsequent second ACL injury. Players with a higher draft pick position were more likely to return to the starting lineup following primary ACL reconstruction. Ultimately, player game utilization and performance following primary ACL reconstruction are not predictive of a subsequent second ACL injury.

## Supplementary Data

ICMJE author disclosure forms
